# No benefits of statins for sudden cardiac death prevention in patients with heart failure and reduced ejection fraction: A meta-analysis of randomized controlled trials

**DOI:** 10.1371/journal.pone.0171168

**Published:** 2017-02-06

**Authors:** Muaamar Al-Gobari, Hai-Ha Le, Mor Fall, François Gueyffier, Bernard Burnand

**Affiliations:** 1 Institute of social & preventive medicine (IUMSP) and Cochrane Switzerland, Lausanne University Hospital (CHUV), Lausanne, Switzerland; 2 Laboratoire de Biologie et Biométrie Evolutive–Service de pharmacologie clinique, Equipe Modélisation des Effets Thérapeutiques (EMET), UMR, Université Claude Bernard Lyon1, Lyon, France; 3 Laboratoire de Pharmacologie & de Pharmacodynamie, Université Cheikh Anta Diop, Dakar, Sénégal; Universita degli Studi di Napoli Federico II, ITALY

## Abstract

**Background and objectives:**

Statins showed mixed results in heart failure (HF) patients. The benefits in major HF outcomes, including all-cause mortality and sudden cardiac death (SCD), have always been discordant across systematic reviews and meta-analyses. We intended to systematically identify and appraise the available evidence that evaluated the effectiveness of statins in clinical outcomes for HF patients.

**Design:**

Systematic review and meta-analysis

**Data sources:**

We searched, until April 28, 2016: Medline, Embase, ISI Web of Science and EBM reviews (Cochrane DSR, ACP journal club, DARE, CCTR, CMR, HTA, and NHSEED), checked clinicaltrials.gov for ongoing trials and manually searched references of included studies.

**Eligibility criteria for selecting studies:**

We identified 24 randomized clinical trials that evaluated the efficacy of statins for HF patients. All randomized clinical trials were assessed for risk of bias and pooled together in a meta-analysis. Pre-specified outcomes were sudden cardiac death, all-cause mortality, and hospitalization for worsening heart failure.

**Results:**

Statins did not reduce sudden cardiac death (SCD) events in HF patients [relative risk (RR) 0.92, 95% confidence interval (CI) 0.70 to 1.21], all-cause mortality [RR 0.88, 95% CI 0.75 to 1.02] but significantly reduced hospitalization for worsening heart failure (HWHF) although modestly [RR 0.79, 95% CI 0.66 to 0.94]. Nevertheless, estimated predictive intervals were insignificant in SCD, all-cause mortality and HWHF [RR, 0.54 to 1.63, 0.64 to 1.19, and 0.54 to 1.15], respectively. An important finding was the possible presence of publication bias, small-study effects and heterogeneity of the trials conducted in HF patients.

**Conclusions:**

Statins do not reduce sudden cardiac death, all-cause mortality, but may slightly decrease hospitalization for worsening heart failure in HF patients. The evaluation of the risk of biases suggested moderate quality of the published results. Until new evidence is available, this study supports the 2013 ACCF/AHA guidelines to not systematically prescribe statins in “only” HF patients, which should help avoid unnecessary polypharmacy.

## Introduction

Heart failure (HF) patients are likely to take more than one drug and tend toward polypharmacy. Guideline-directed medical therapy includes angiotensin converting enzyme inhibitors, beta-blockers, aldosterone antagonists as well as implantable cardioverter defibrillators, which all have reported a reduction in mortality and morbidity in heart failure patients [1–4]. Though, such benefits are still insufficient to the current management need as almost half of HF patients die within 5 years after initial diagnosis and half of the mortality is attributed to sudden cardiac death (SCD) [5,6]. More potential benefits are hypothesized with statin treatment but current ACCF/AHA guidelines do not recommend statins for only HF diagnosis [7]. However, 3-hydroxy-3-methylglutaryl coenzyme A (HMG-Co A) reductase inhibitors or simply statins are still widely prescribed for HF patients [8].

Several studies [9–11] evaluated the effects of statin on sudden cardiac death prevention but with a variety of population characteristics which made the result difficult to apply for HF patients. Oppositely, two large randomized clinical trials (RCTs) [12,13] in heart failure reported no reduction of all-cause mortality and SCD events by statins. Moreover, studies often evaluated surrogate endpoints or biomarkers other than important clinical endpoints such as mortality and that might have exaggerated the expected benefits of statins[14].

A systematic review [15], published in 2006, stressed on the importance of this research question and pointed out the conflicting and unclear evidence. CORONA [13] and GISSI-HF [12] (unpublished at that time) was expected to resolve the issue. In the contrary, both studies, after publication, raised controversial statements and debates. The morbidity and mortality rate among HF patients is considerably high and an emphasis on effective prevention strategies would lead to a significant reduction of such events. Similarly, HF patients have a reduced longevity thus the need for providing clinicians and health care actors an optimal evidence-based strategy is of vital importance.

Nevertheless, current trials, systematic reviews and meta-analyses [16–24] for statins have shown mixed results for major HF outcomes. Positive studies were not immune to bias, serious limitations or indirectness. Therefore, we intended to evaluate and update the quality of evidence of statins efficacy to reduce SCD, mortality or hospitalization for worsening heart failure (HWHF) by means of a systematic review and a meta-analysis with a careful consideration of potential biases in published studies.

## Methods

### Study search strategy

**Fig 1:**
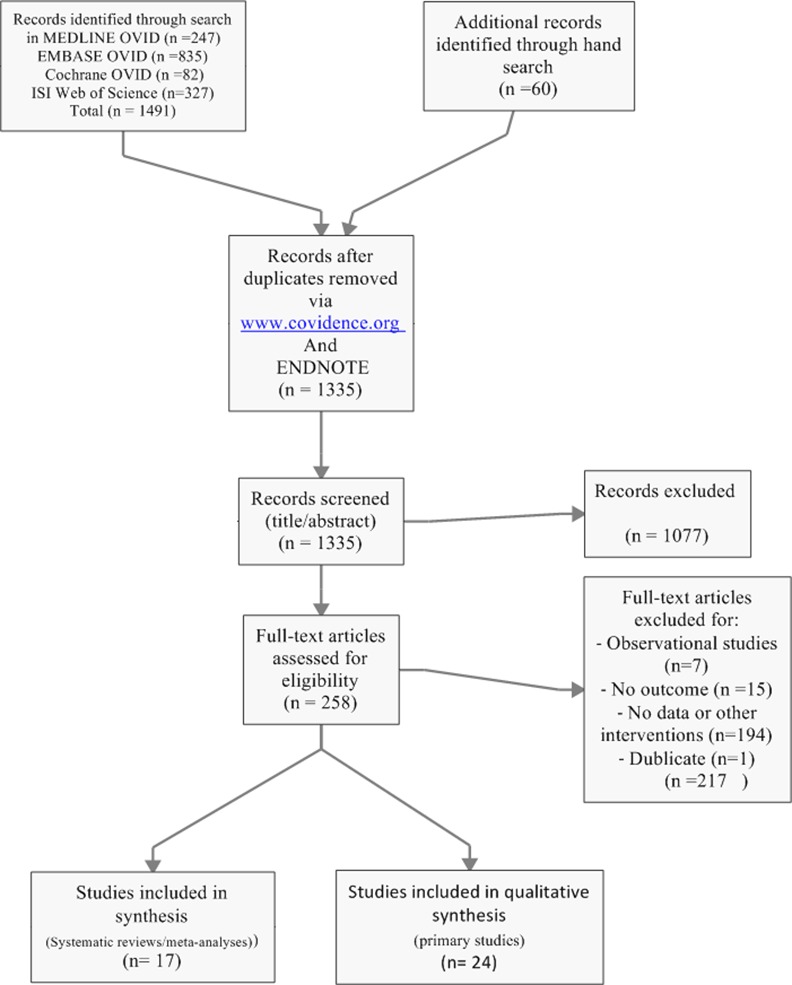
Flow chart for search result.

We searched Medline (1946 to April 28, 2016), Embase (1974 to April 28, 2016), EBM reviews (Cochrane DSR, ACP journal club, DARE, CCTR, CMR, HTA, and NHSEED) (to April 28, 2016), and ISI web of science (‘‘All years” to April 28, 2016) via an Ovid online interface and identified systematic reviews and meta-analyses via a search strategy accessible on S1 File. In a first step, we used a filter [25,26] to search for systematic reviews and meta-analyses and initially excluded individual clinical trials for the purpose of our study. In a second step, we searched for primary studies and included randomized clinical trials evaluating statins in heart failure patients. In Medline and Embase, we combined medical subject heading terms (MeSH and EMTREE respectively), text words as well as a truncation when appropriate. The method included a combination of a disease (i.e., heart failure), an intervention (i.e., statins) as well as the aforementioned filter. Also, we added an outcome (i.e., sudden cardiac death and/or mortality) to limit the research output. No language restrictions were applied and a bimonthly alert was set up for an automatic update during the study. We also checked the references of included studies for potential additional studies, searched clinicaltrials.gov and tried to contact authors for additional or missing data. Fig 1 shows the search strategy results according to the PRISMA guidelines (see also S2 File).

### Selection criteria and data abstraction

We included randomized clinical trials (RCTs) that evaluated statins efficacy in heart failure patients and contained at least one outcome of interest. No exclusion was based on treatment duration or follow-up period, language or *intention-to-treat* analysis. Risk of bias was determined by the Cochrane risk bias assessment tool [27]. Two reviewers (MA&HHL) independently collected data and were checked by a third reviewer (MF) while discrepancies were resolved via discussion and consensus. Abstracted data included eligibility criteria, baseline characteristics, study design (including treatment and control arms), follow-up duration, and outcomes. Pre-specified outcomes of interest included sudden cardiac death (SCD), all-cause mortality and hospitalization for worsening heart failure (HWHF) and were analyzed according to an *intention-to-treat* principle when possible.

### Statistical analysis

We pooled the data in a meta-analysis, using random-effects and fixed-effects model with Mantel-Haenszel methods that are preferable to inverse variance methods in case of few events [27]. Sensitivity and subgroup analyses were done to verify the extent to which different hypotheses might change our confidence in the result. The effect size was relative risk or risk ratio (RR), confidence intervals (CIs) of 95% was given when applicable and a significance level was determined at two-sided alpha less than 5%. We reported both fixed-effect and random-effects models for the meta-analysis and used risk ratios (RR) as the summary effect estimate that may give better interpretation as the outcome is hospitalization or death events, termed bad outcome. We calculated the absolute effect or the absolute risk reduction (ARR) for SCD, all-cause mortality and HWHF with an assumed control risk (ACR) of 0.11, 0.27 and 0.38 respectively. To check the validity of choosing the summary statistic, odds ratio (OR) was used instead of RR in a sensitivity analysis. The 95% prediction interval, an interval that estimate a treatment effect of a new or future trial within which we are confident it lies, was given when a random-effects model is used. Also, we rated the quality of the evidence using a summary of findings table from the GRADE approach [28]. We computed the sample size required to reach a statistical significance according to the observed differences between the groups of statin and placebo or control for all outcomes [α = 5% and power (1- β) = 0.80] and intended to stratify the studies according to follow-up and sample size in subgroup and sensitivity analyses. Heterogeneity was measured by I-squared (I^2^, variation in RR/OR attributable to heterogeneity) and Tau-squared (Tau^2^, estimate of between-study variance). We noticed that the follow-up period differed largely from one study to another from one month [29,30] to more than 30 months [12,13,31]. Therefore, studies were grouped according to follow-up as a potential determinant of any heterogeneity among studies. Data was analyzed with STATA version 14.1 (StataCorp LP, Texas), RevMan (version 5.3) and GradePro (version 3.6.1).

## Results

### Study selection

Electronic databases and manual searches resulted in 1335 studies after removal of duplicates. After screening of titles and abstracts and examination of selected full-texts, we ended up with 17 systematic reviews/meta-analyses [9,11,18–24,32–39] and 24 randomized controlled trials (RCTs) [12,13,40–51] [29,31,52] [30,53–58] (Fig 1).

### Study and baseline patient characteristics

As shown in Tables 1 and 2, we identified 24 RCTs of statins in heart failure (HF) patients for inclusion in this meta-analysis, which enrolled a total of 11,463 patients. The mean and the median of follow-up duration were 11.5 and 6 months respectively. The studies included in majority male participants (ratio ranged from 54% to 100%) with an average age of 60 years. The mean age of the three biggest studies (CORONA [13], GISSI-HF [12] and Takano H. et al. (PEARL) [31]) was 68 years. All studies included HF patients with New York Heart Association classification (NYHA) ranging from I-IV and at least 9 studies included stable HF patients. Seven studies included non-ischaemic HF patients, 4 studies exclusively ischaemic, 12 included both types and one unknown. All patients in included studies had an ejection fraction less than 45% and no one included HF with a preserved ejection fraction (HFpEF). Baseline mean lipid levels were relatively similar from one study to another with mean low-density lipoprotein cholesterol (LDL-c) of 3.3 for 19 studies and mean total cholesterol (TC) of 5.19 for 3 studies and unknown for 2 studies. Only 5 studies[13] [12] [31] [58] [54] had a mortality endpoint while others evaluated surrogate endpoints and biomarkers. Statins were compared to placebo in 13 studies and to no statin in 10 studies; one study compared two different doses of statins to a no statin group[56].

**Table 1:** Randomized trials of statins in heart failure patients.

Trial [Reference]	Publication Year	Number of patients (statin/comparator)	Name of drug	Comparator	Mean baseline Lipids	Primary Endpoint	Dosage (mg/day)	Follow-up (months)
AbdulHul E. et al.[40]	2012	56(28/28)	Atorvastatin	No statin	LDL-c = 2.73 mmol/L	Biomarkers, BNP	40	6
							
Bielecka-D. et al.[41]	2009	68(41/27)	Atorvastatin	No statin	LDL-c = 3.15 mmol/L	Factors affect HWHF/mortality	10–40	6
Bleske BE. et al.[42]	2006	18(9/9)*	Atorvastatin	Placebo	LDL-c = 2.84 mmol/L	Surrogate markers in NICM	80	3
CORONA [13]	2007	5011(2514/2497)	Rosuvastatin	Placebo	LDL-c = 3.55 mmol/L	HWHF/SCD/mortality	10	32.8
Erbs S.et al. [43]	2011	42(22/20)	Rosuvastatin	Placebo	LDL-c ≥ 3.78 mmol/L	Pleiotropic effects	40	24
Gissi-HF Investigators [12]	2008	4574(2285/2289)	Rosuvastatin	Placebo	NR	HWHF/SCD/mortality	10	46.8
Hammad A. et al. [44]	2005	23(13/10)	Atorvastatin	Placebo	TC ≈ 3.5 mmol/L	Autonomic nervous system	40	3
Hong et al.[58]	2005	202(106/96)	Simvastatin	No statin	LDL-c ≈ 3.62 mmol/L	PCI rate, restenosis, mortality	40	12
Horwich TB. et al. [45]	2011	26(14/12)	Atorvastatin	Placebo	LDL-c ≈ 2.79 mmol/L	Sympathetic nervous system	10	3
Krum et al. [46]	2007	86(40/45)	Rosuvastatin	Placebo	NR	Ventricular remodeling	10–40	6
Laufs U. et al.[47]	2004	15(8/7)	Cerivastatin	Placebo	LDL-c ≈ 3.46 mmol/L	Pleiotropic effects	0.4	5
Liu M. et al. [48]	2009	64(32/32)	Atorvastatin	Placebo	LDL-c ≈ 3.03mmol/L	Inflammation	10	3
Mozaffarian D.et al. [49]	2005	22(12/10)	Atorvastain	Placebo	LDL-c = 3.33 mmol/L	Inflammation	10	2
Node K. et al. [50]	2003	51(23/25)	Simvastatin	Placebo	LDL-c ≈ 3.85 mmol/L	Cardiac function	5–10	3.5
Sola S. et al. [51]	2006	108(46/43)	Atorvastatin	Placebo	LDL-c ≈ 3.12 mmol/L	Inflammation	20	12
Strey CH. et al. [52]	2006	23(11/12)*	Atorvastatin	Placebo	LDL-c ≈ 3.56 mmol/L	Endothelial function	40	1.5
Takano H. et al.[31]	2013	577(288/286)	Pitavastatin	No statin	LDL-c ≈ 3.24 mmol/L	Mortality /HWHF/stroke	2	35.5
Tousoulis D.et al.[29]	2005	38(19/19)	Atorvastatin	No statin	TC ≈ 4.83 mmol/L	Inflammation	10	1
Tousoulis D.et al.[30]	2004	38(14/12)	Atorvastatin	No statin	TC ≈ 5.04 mmol/L	Inflammation	10	1
Vrtovec B. et al. [53]	2005	80(40/40)	Atorvastatin	No statin	TC ≈ 5.07 mmol/L	HRV/ QTV/ QTc	10	3
Vrtovec B. et al. [54]	2008	110(55/55)	Atorvastatin	No statin	LDL-c = 2.45 mmol/L	SCD	10	12
Wojnicz R. et al. [55]	2006	74(36/38)	Atorvastatin	No statin	LDL-c ≈ 4.18 mmol/L	Inflammation	40	6
Xie RQ. et al.[56]	2008	119(78/41)	Atorvastatin	Statin/no statin	LDL-c ≈ 3.64 mmol/L	Cardiac function	10–20	12
Yamada T. et al.[57]	2007	38(19/19)	Atorvastatin	No statin	LDL-c ≈ 3.02 mmol/L	Cardiac function	10	31

BNP: B-type natriuretic peptide; HRV: heart rate variability; QTV: QT variability; QTc: QTc interval; HWHF: Hospitalization for worsening heart failure; LDL-c: lowdensity lipoprotein cholesterol; NICM: Non-Ischaemic Cardiomyopathy; PCI: percutaneous coronary intervention; SCD: sudden cardiac death; TC: total cholesterol; NR: not reported; ≈: approximately.

* Estimated values

**Table 2:** Patient characteristics in randomized trials of statins in heart failure patients.

Trial [Reference]	Mean age (Years)	Male (%)	Inclusion criteria	Population (Ischaemic or non-ischaemic, %)	Mean LVEF (%)	NYHA
AbdulHul E. et al.[40]	72	68	Mild-moderate HF, LVEF ≤ 45%	Ischaemic, 64	35	II-III
Bielecka-D. et al.[41]	57	85	Stable HF with DC	NR	29	I-III
Bleske BE. et al.[42]	56	60	CHF due to NICM, LVEF<40%	Non-ischaemic	25	I-III
CORONA [13]	73	76	Ischaemic HF, age ≥ 60, LVEF < 40%	Ischaemic,100	31	II-IV
Erbs S.et al. [43]	62	76	CHF—Ischaemic HF or DC	Ischaemic,28	30	II-III
Gissi-HF Investigators [12]	68	77	CHF, No LVEF restriction	Ischaemic,40	33	II-IV
Hammad A. et al. [44]	67	86	Stable systolic HF	Ischaemic, 57	31	II-III
Hong et al.[58]	61	72	Patients underwent PCI for AMI	Ischaemic, 100	31	II-IV
Horwich TB. et al. [45]	48	62	Symptomatic HF, LVEF ≤ 35%	Non-ischaemic	26	I-III
Krum et al. [46]	62	80	NYHA II-IV, LVEF ≤ 35% or ≤ 40%	Ischaemic,12	29	II-IV
Laufs U. et al.[47]	51	NR	CHF with NICM	Non-ischaemic	42	II-III
Liu M. et al. [48]	67	94	NYHA II-III, CHF with IDCM	Ischaemic/ Non-ischaemic	20	II-IV
Mozaffarian D.et al. [49]	51	86	Ambulatory HF, LVEF < 40%	Ischaemic, 9	31	II-III
Node K. et al. [50]	54	69	HF with IDCM	Non-ischaemic	33	II-III
Sola S. et al. [51]	54	63	Stable non-ischaemic HF	Non-ischaemic	33	II-IV
Strey CH. et al. [52]	61	70	Symptomatic HF, LVEF < 40%	Non-ischaemic	30	II-III
Takano H. et al.[31]	63	81	CHF, LVEF ≤ 45%	Ischaemic, 27	34	II-III
Tousoulis D.et al.[29]	69	100	Ischaemic HF, LVEF ≤ 35%	Ischaemic	25	III-IV
Tousoulis D.et al.[30]	58	NR	Stable HF. LVEF ≤ 35%	Ischaemic, 65.7	25	II-IV
Vrtovec B. et al. [53]	67	54	Stable HF, LVEF < 30%	Ischaemic, 62	25	III
Vrtovec B. et al. [54]	62	61	Stable HF, LVEF < 30%	Ischaemic, 59	25	III
Wojnicz R. et al. [55]	38	81	Stable HF with DC	Non-Ischaemic	28	II-III
Xie RQ. et al.[56]	NR	NR	Ischaemic HF, LVEF<45%	Ischaemic,100	38	II-IV
Yamada T. et al.[57]	64	79	Stable HF, NYHA I-III	Ischaemic, 53	34	I-III

AMI: acute myocardial infarction; CHF: Chronic Heart Failure; DC: dilated cardiomyopathy; DCM: dilated cardiomyopathy; HF: heart failure; IDCM: idiopathic dilated cardiomyopathy; LVEF: Left Ventricular Ejection Fraction; NICM: Non-Ischaemic Cardiomyopathy; NR: not reported; NYHA: New York Heart Association; PCI: percutaneous coronary intervention.

### Risk of bias and quality of studies

**Fig 2:**
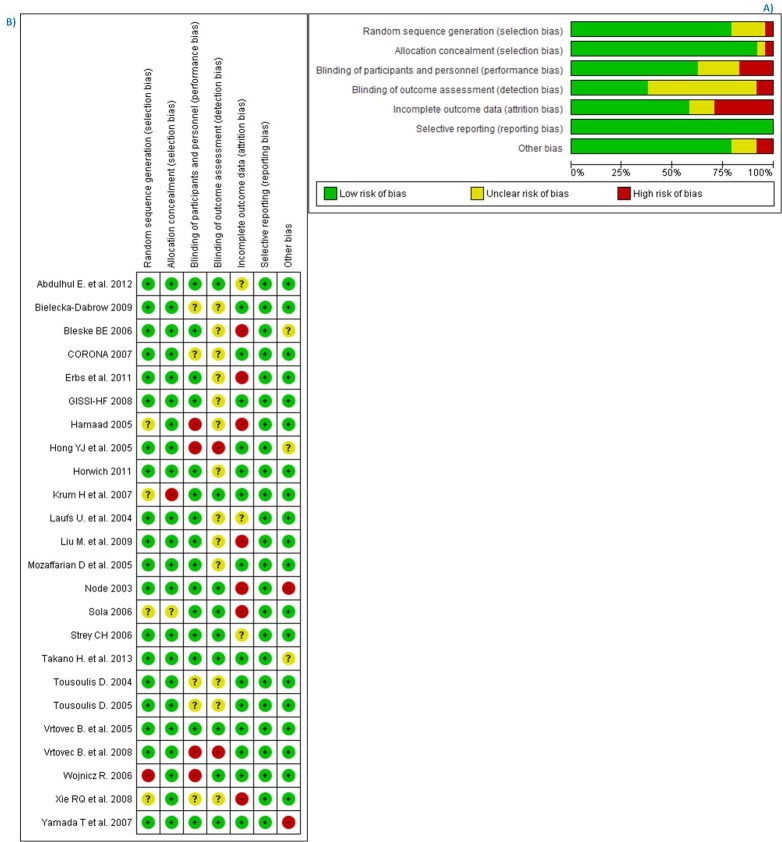
Risk of bias for included studies. A) Risk of bias graph for statin trials in heart failure patients: review authors' judgments about each risk of bias item presented as percentages across all included studies. B) Risk of bias summary for statins in heart failure patients: review authors' judgments about each risk of bias item for each included study.

**Fig 3:**
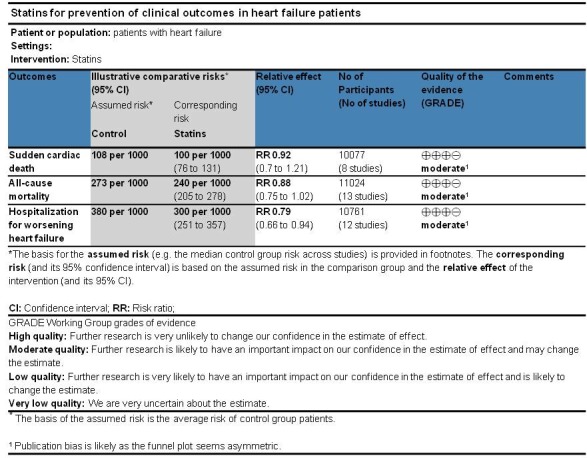
GRADE summary of findings table.

We assessed studies’ risk of bias by means of the Cochrane bias tool[27]. We noticed that the *intention-to-treat* principle was not followed in all trials, among which 10 had either high or unclear risk (Fig 2(B)). Most of the included trials had unclear risk towards blinding of investigators or outcome assessors. Overall, the studies fluctuated from high to moderate/low level of quality. Fig 2 (A) shows the summary of bias risk assessment in percentages and for each included trial. We also rated each outcome (SCD, All-cause mortality and hospitalization for worsening heart failure (HWHF)) according to the GRADE approach (Fig 3). Studies were evaluated per outcome for any bias, inconsistency, indirectness, imprecision or publication bias. The latter was a concern for all outcomes and this resulted in moderate evidence. Due to missing data or absence of events, only 8 studies for SCD, 13 studies for all-cause mortality and 12 studies for HWHF were analyzed.

### Efficacy of statins and synthesis of results

**Fig 4:**
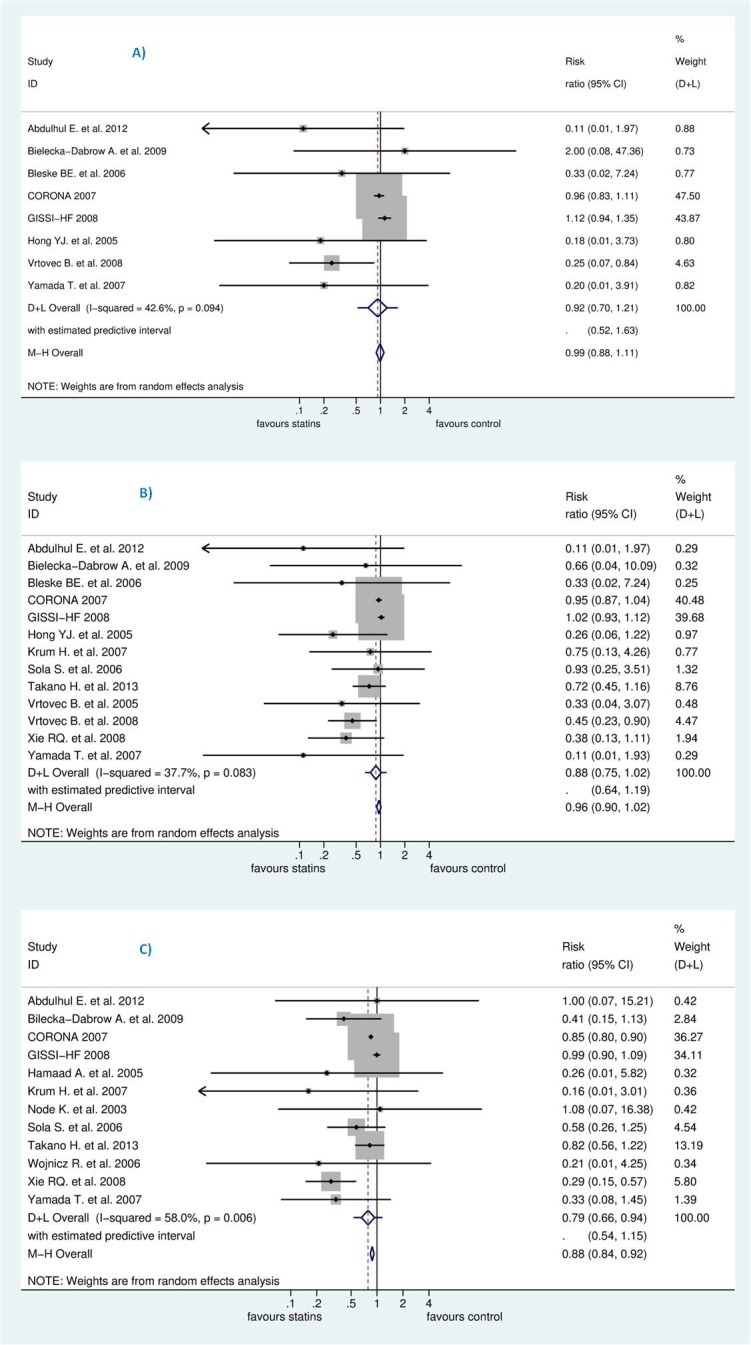
**Efficacy of statins compared with control in heart failure for the prevention of (A)** sudden cardiac death (SCD) **(B)** all-cause mortality, and **(C)** hospitalization for worsening heart failure (HWHF).

We pooled the studies to evaluate effects of statins on the reduction of sudden cardiac death (SCD), all-cause mortality, and hospitalization due to worsening heart failure (HWHF). The forest plots in Fig 4 shows insignificant reduction in SCD [Risk Ratio (RR) 0.92; 95% CI, 0.70 to 1.21, P = 0.554] and all-cause mortality [RR 0.88; 95% CI, 0.75 to 1.02, P = 0.092] and a statistically significant difference in HWHF [RR 0.79; 95% CI, 0.66 to 0.84, P = 0.008]. Random-effects and fixed-effect models were used to quantify the summary statistic by RR. The effect size of fixed-effects models was quite similar to CORONA [13] which weighted 59.9%, 50.8%, and 63.7% for the respective outcomes of the study population.

In Grade output (Fig 3), the SCD rate was 10.7% (n = 540/5057) in those treated with statins compared with 10.8% (n = 544/5020) in those treated with placebo/control with an absolute effect of 9 fewer per 1000 (from 33 fewer to 23 more). All-cause mortality rate was 25.9% (n = 1436/5549) in those treated with statins compared with 27.3% (n = 1494/5475) in those treated with placebo/control with an absolute effect of 33 fewer per 1000 (from 68 fewer to 5 more). Also, hospitalization rate was 33.33% (n = 1804/5412) in those treated with statins compared with 38% (n = 2033/5349) in those treated with an absolute effect of 80 fewer per 1000 (from 23 fewer to 129 fewer) (see also S1 Table). The estimated sample size needed to detect a statistically significant difference, given the effect size found, were 599506, 15394 and 1642 respectively for SCD, all-cause mortality and HWHF [59]. Moreover, the estimated predictive intervals (PIs) were statistically insignificant [0.52 to 1.63, 0.54 to 1.15, and 0.64 to 1.19] in SCD, all-cause mortality and HWHF, respectively, as shown in Fig 4.

The retrieved published systematic reviews and meta-analyses were evaluated against potential biases and are compared with the results of our systematic review in the discussion section.

### Meta-regression and heterogeneity of combined studies

For SCD, all-cause mortality and HWHF, heterogeneity estimators were, respectively: I^2^ = (42.8%, 37.9%, and 58%) and Tau^2^ = (0.0353, 0.0135, and 0.0215). We intended to explain the heterogeneity by a meta-regression analysis for cholesterol levels changes for each outcome but this was not statistically feasible because of zero events in the intervention or control group in some trials [log (OR) or log (RR) became undefined]. Also, at least because the number of studies left for any outcome assessment decreased and the risk of type Ι error may have probably increased.

**Fig 5:**
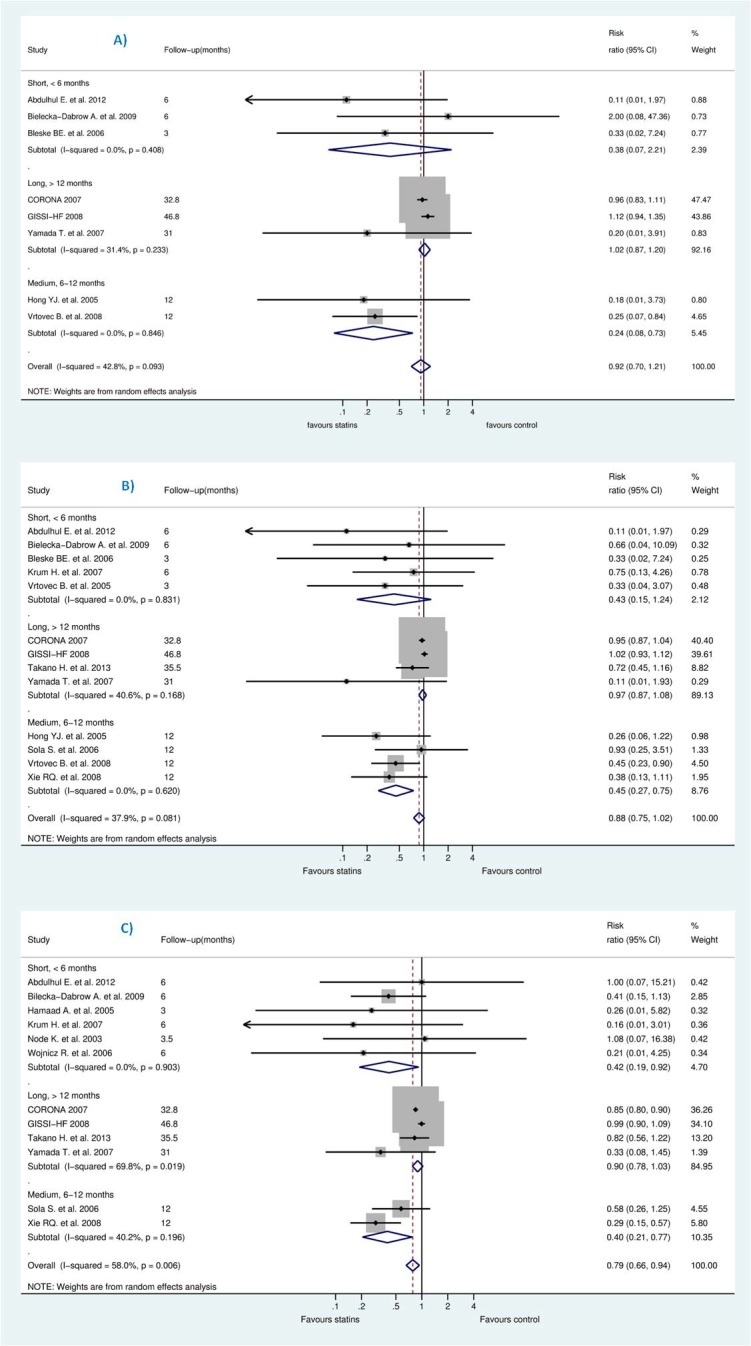
**Efficacy of statins compared with control in heart failure stratified by follow-up duration for the prevention of (A)** sudden cardiac death (SCD) **(B)** all-cause mortality, and **(C)** hospitalization for worsening heart failure (HWHF).

However, we tried to explain the heterogeneity observed for assessed outcomes by stratification according to follow-up duration. As shown in Fig 5, studies were classified into three categories: 6 months or less, more than 6 months and less than 12, and more than 12 months. Negative result was maintained in this analysis except for studies of less than 12 months of follow-up which was likely due to small-study effects, revealed by insignificant reduction for studies more than 12 months which represented between 85–92% of the studied population.

### Sensitivity and subgroup analyses

**Fig 6:**
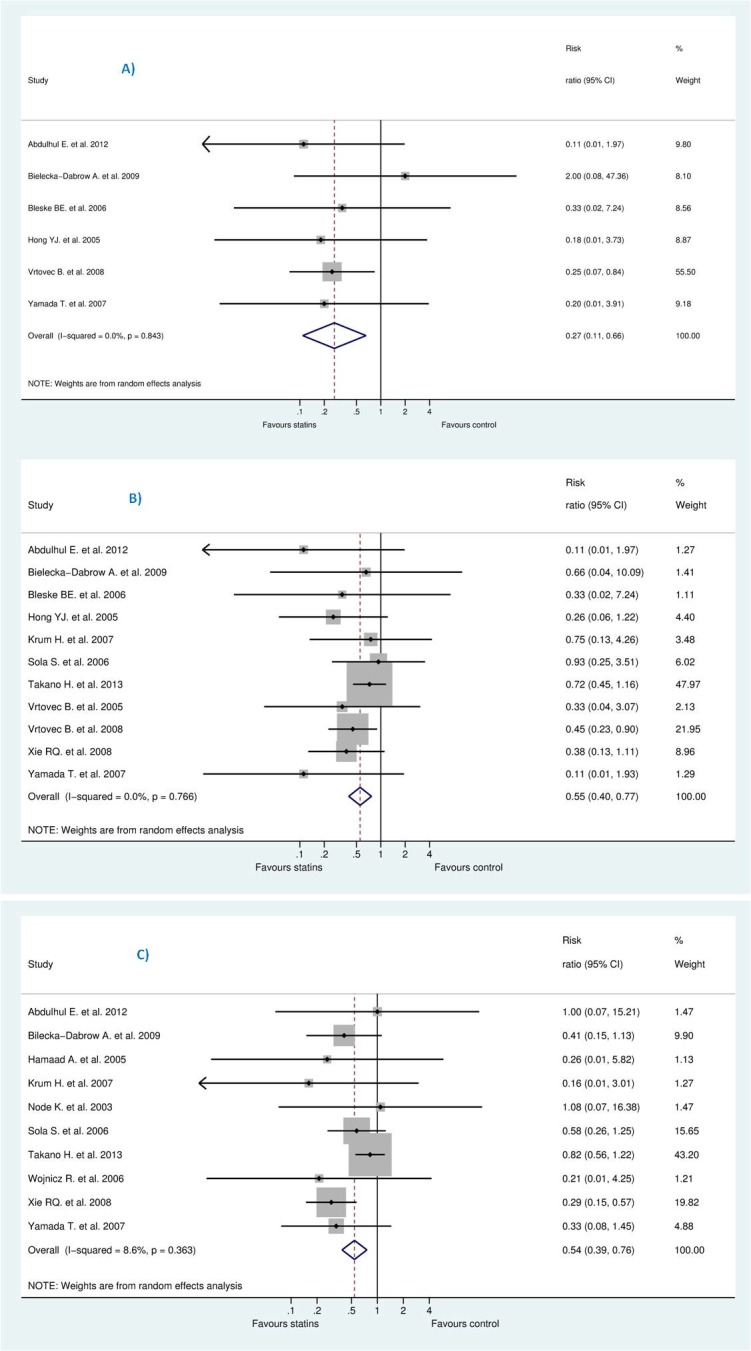
Sensitivity analysis without corona and GISSI-HF.

Both CORONA [13] and GISSI-HF [12] comprised at least 70% of the study population irrespective of the outcome or the model used. Therefore, we conducted a sensitivity analysis to assess the impact of these trials on the results by excluding them from the random-effect estimate. This resulted into significant difference: RR for SCD [0.27 (95% CI, 0.11 to 0.66), P = 0.004], RR for all-cause mortality [0.55 (95% CI, 0.40 to 0.77), P = 0.0001], and RR for HWHF [0.54 (95% CI, 0.39 to 0.76), P = 0.0001] (see Fig 6).

**Fig 7:**
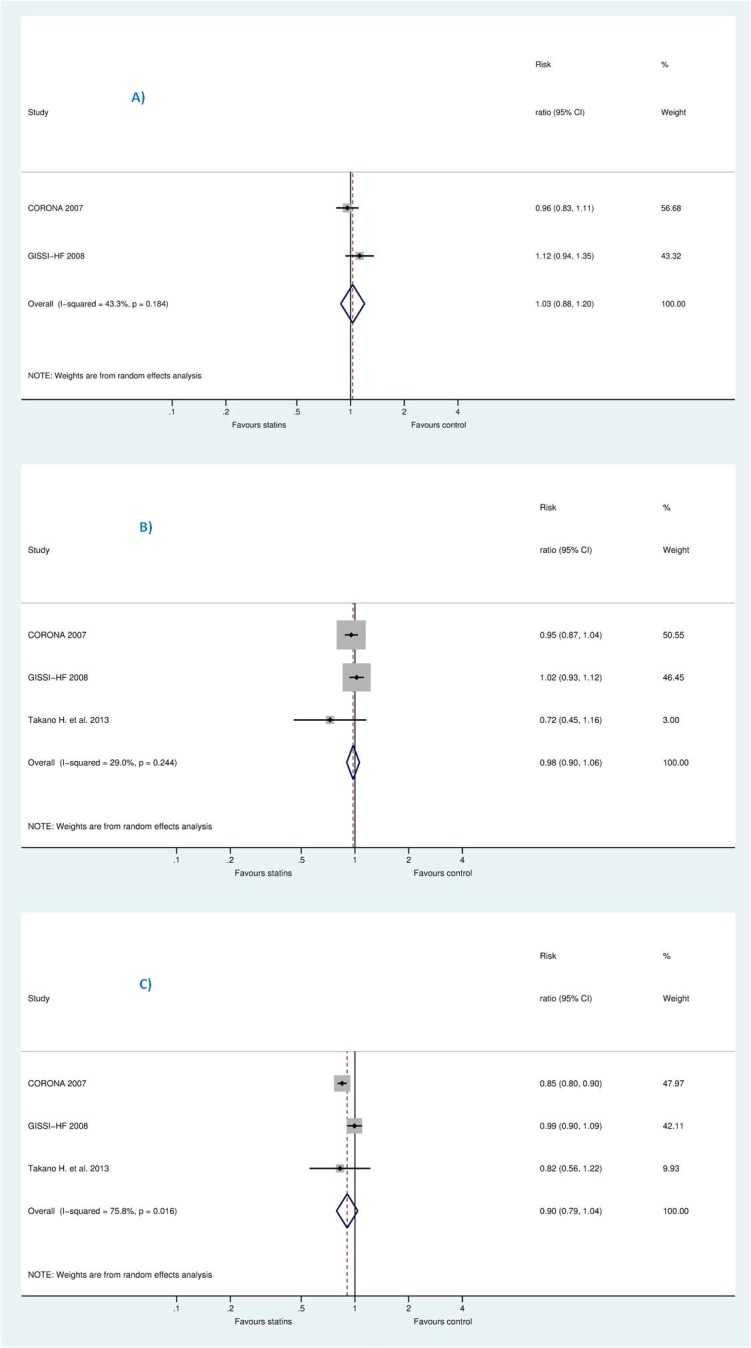
Sensitivity analysis without less powered studies.

**Fig 8:**
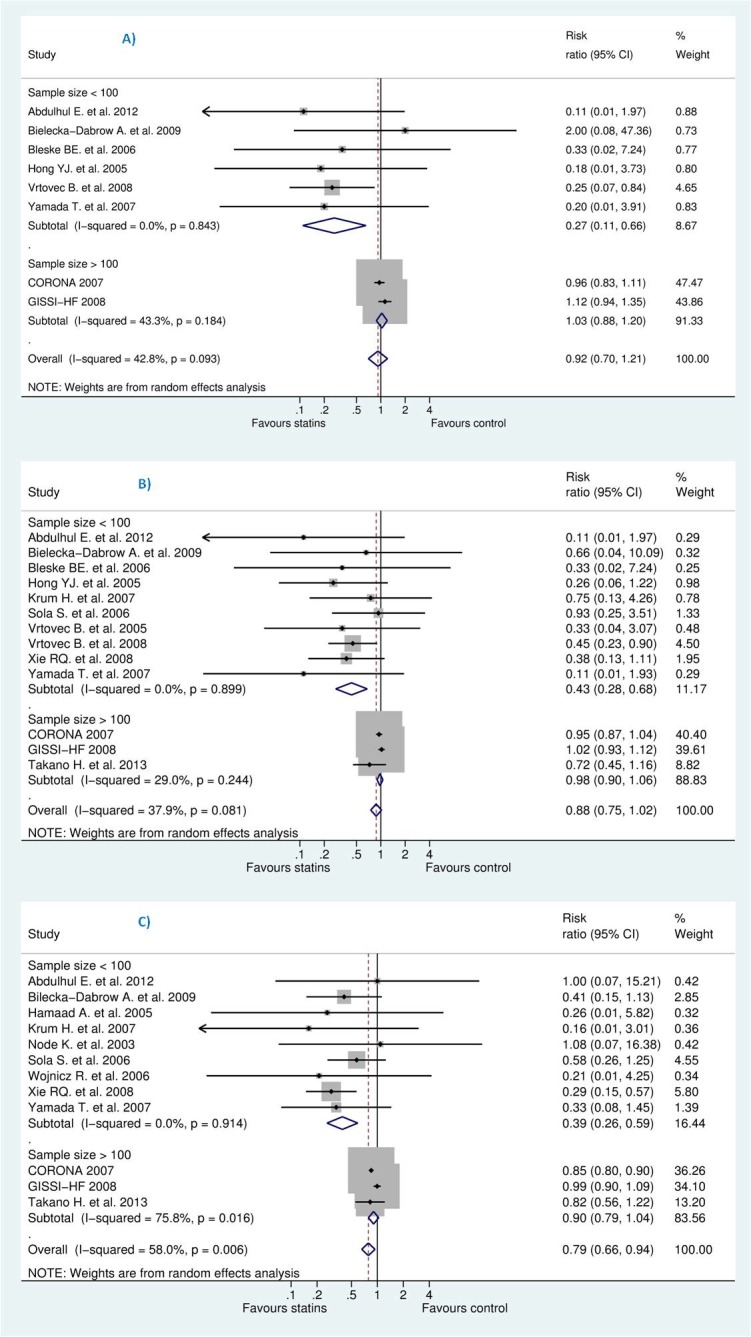
**Efficacy of statins compared with control in heart failure stratified by sample size (more than 100 or less than 100) for the prevention of (A)** sudden cardiac death (SCD) **(B)** all-cause mortality, and **(C)** hospitalization for worsening heart failure (HWHF).

On the other hand, after deleting less powered studies, i.e., those with less than 100 patients in each group, this resulted into insignificant increase in SCD [RR 1.03 (95% CI, 0.88 to 1.20), P = 0.725], RR for all-cause mortality [0.98 (95% CI, 0.90 to 1.06), P = 0.566], and RR for HWHF [0.90 (95% CI, 0.79 to 1.04), P = 0.149]. The remaining studies were (CORONA [13], GISSI-HF [12] for SCD plus Takano H. et al. (PEARL) [31] for all-cause mortality and HWHF outcomes) (see Figs 7 and 8).

**Fig 9:**
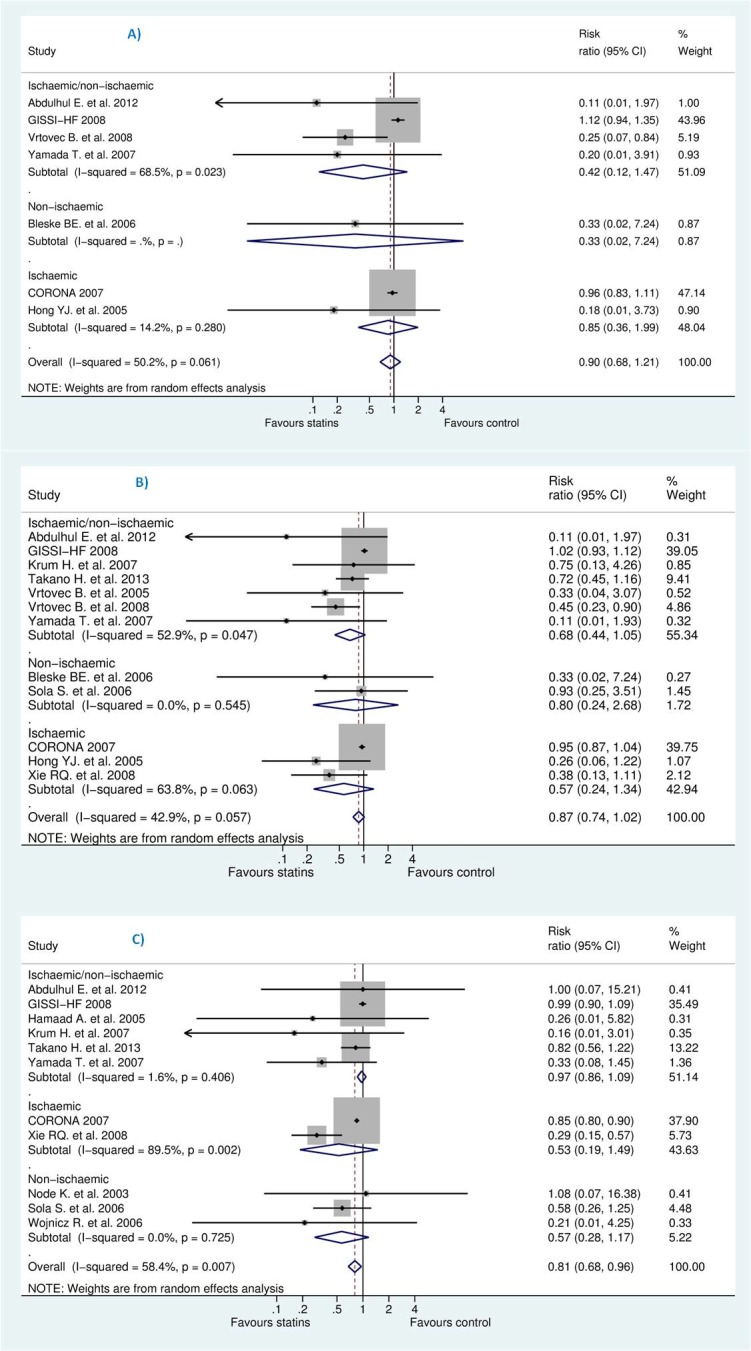
**Efficacy of statins compared with control in heart failure stratified by population type (ischaemic or non-ischaemic) for the prevention of (A)** sudden cardiac death (SCD) **(B)** all-cause mortality, and **(C)** hospitalization for worsening heart failure (HWHF).

**Fig 10:**
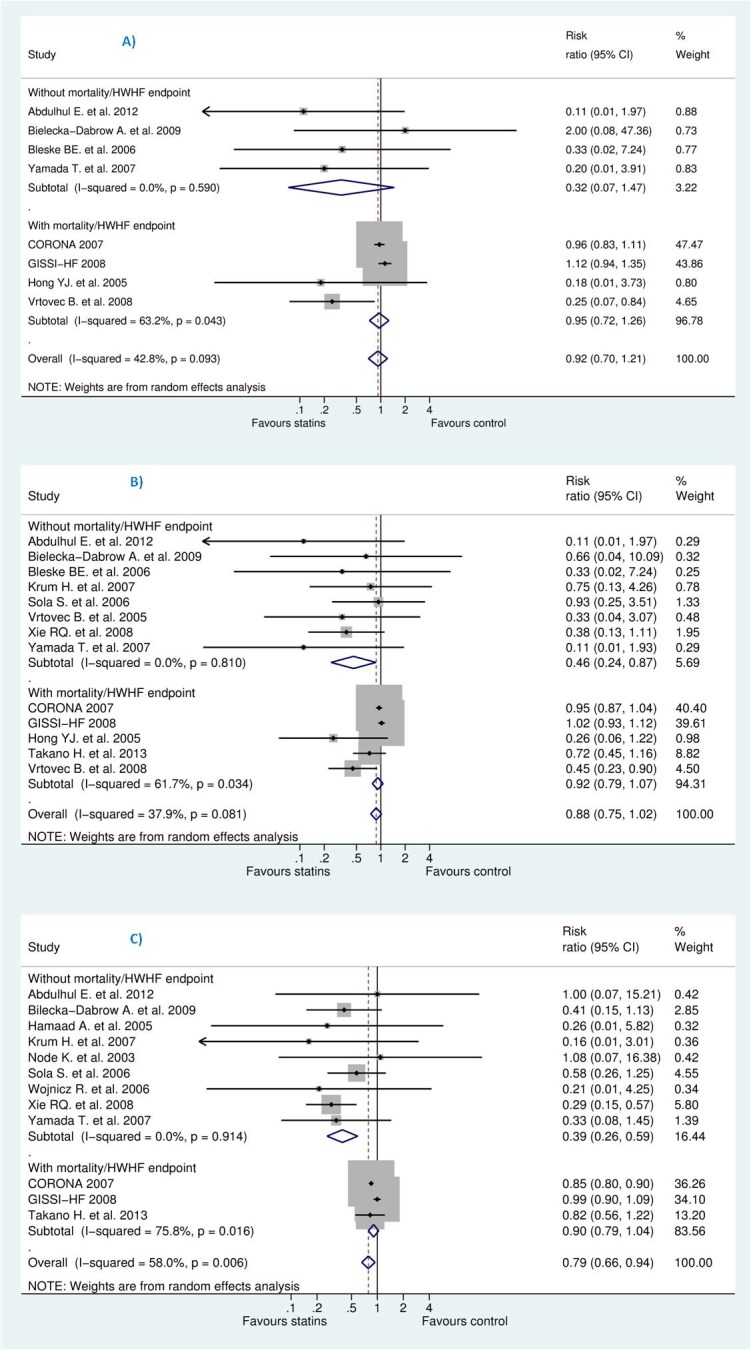
**Efficacy of statins compared with control in heart failure stratified by endpoint (those with against without mortality and/or HWHF) for the prevention of (A)** sudden cardiac death (SCD) **(B)** all-cause mortality, and **(C)** hospitalization for worsening heart failure (HWHF). [Random-effects model].

To test our choice of the summary statistic, we shifted to OR instead of RR, the result changed slightly: OR for SCD [0.89 (95% CI, 0.65–1.23), P = 0.49], OR for all-cause mortality [0.81 (95% CI, 0.65–1.00), P = 0.05] and OR for HWHF [0.66 (95% CI, 0.50 to 0.87), P = 0.008]. Also, we stratified studies according to being ischaemic, non-ischaemic or both (see Fig 9). This resulted into statistically insignificant difference for all outcomes: SCD, all-cause mortality and HWHF. The effect size for ischaemic group, non-ischaemic and ischaemic/non-ischaemic were, respectively: SCD [RR 0.42 (95% CI, 0.12–1.47), 0.33 (95% CI, 0.02–7.24), and 0.85 (95% CI, 0.36–1.99), overall P = 0.49]; all-cause mortality [RR 0.68 (95% CI, 0.44–1.05), 0.80 (95% CI, 0.24–2.68), and 0.57 (95% CI, 0.24–1.34), overall P = 0.087]; HWHF [RR 0.97 (95% CI, 0.86–1.09), 0.53 (95% CI, 0.19–1.49), and 0.57 (95% CI, 0.28–1.17), overall P = 0.015]. We noticed that only five studies had our outcome of interest as a primary endpoint. When studies were stratified according to their respective endpoints, this resulted into significant differences and a considerable heterogeneity among studies with mortality and/or hospitalization endpoints (Fig 10).

### Publication bias

**Fig 11:**
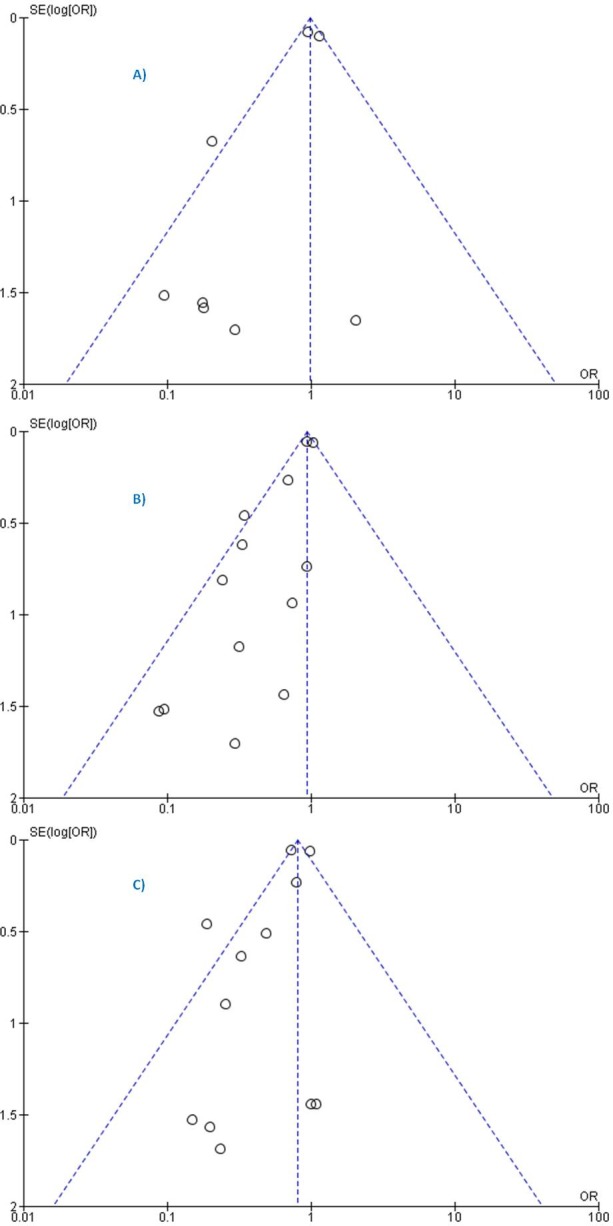
**Funnel plots of SE (log odds ratio) by odds ratio to evaluate publication bias for the effect of treatment for prevention of (A)** sudden cardiac death (SCD) **(B)** all-cause mortality, and **(C)** hospitalization for worsening heart failure (HWHF). (Fixed-effects model).

According to the funnel plots, we estimated that publication bias was likely for the three outcomes (SCD, all-cause mortality, and HWHF); asymmetry appeared bigger for all-cause mortality (Fig 11). Funnel plot analyses were reinforced [60] for all-cause mortality and HWHF (for which the number of trials was above 10) by Egger and Harbord tests which showed significant small-study effects for all-cause mortality (P = 0.001) but insignificant result for HWHF (P = 0.088 and P = 0.062). Overall, publication bias was taken into account in the GRADE evaluation and the synthesis of the study results.

## Discussion

Our systematic review and meta-analysis showed no clinical benefits of adding statins to the treatment of HF patients with reduced left ventricular ejection fraction. Statins are ineffective for the prevention of sudden cardiac death (SCD), all-cause mortality but may slightly decrease hospitalization for worsening heart failure (HWHF). However, the apparent reduction of HWHF might result from small-study effects. Actually the studies with a longer follow-up (more than one year), represented almost 85% of the population in this analysis, showed insignificant reduction. The little reduction in the number of hospitalization is not supported by the estimated predictive intervals [0.64 to 1.19] derived from random effects models.

Several previously published meta-analyses [9,11,18,38] evaluated the effects of statins in SCD prevention and reported a decrease but all studies were limited by indirectness as they included different populations, i.e. patient with coronary heart disease (CHD), recent and history of myocardial infarction (MI), or diabetes, and even primary-and-secondary prevention statin trials [18].

Another meta-analysis [24] claimed that statins reduce all-cause mortality in chronic heart failure. The study had a biased result as authors lumped together randomized clinical trials (RCTs) with non-causal observational studies [61] with a high heterogeneity (I^2^ = 90%).

Our sensitivity and subgroup analyses suggested a potential publication bias, as indicated by the unbalanced presence of small studies which showed beneficial effects and heterogeneity among included studies. The two large clinical trials CORONA [13] and GISSI-HF [12] showed no benefit of statins and thus different results than the small studies. Many argued that CORONA [13] and GISSI-HF [12] used a hydrophilic statin (rosuvastatin) that may have a different effectiveness than the predominantly lipophilic statins (atorvastatin, simvastatin, lovastatin, fluvastatin, cerivastatin and pitavastatin)—pravastatin and rosuvastatin are relatively hydrophilic[62].

Based on statin type, two meta-analyses [17,21] included lipophilic statins and stated that they decreased all-cause mortality, cardiovascular death and HWHF. Both studies included only 13 RCTs, compared to 24 in this analysis, and the latter mistakenly reported death events in Hammad A. et al. [44] and Node K. et al [50] whereas the authors of those small trials stated that no death occurred during the study period. This limitation created more small-scale trials that exaggerated the benefits of lipophilic statins In addition, those meta-analyses included *Takano H*. *et al*. [31], representing at least 48% of the population in the analysis, which used a lipophilic statin (pitavastatin) and reported no overall significant decrease in all-cause death or HWHF.

Moreover, a meta-analysis [63] pooled 10 trials (out of the 24 in our analysis) and showed no benefits for overall non stratified population and concluded for trend toward benefits for atorvastatin at subgroup analysis. However, given the influence of small, poor quality RCTs on the overall pooled results and only 5% of the studied population for atorvastatin, the authors' conclusion seemed overstated [64]. Such result was quite similar to a study [22] which excluded both CORONA [13] and GISSI-HF [12] for no apparent reason.

**Fig 12:**
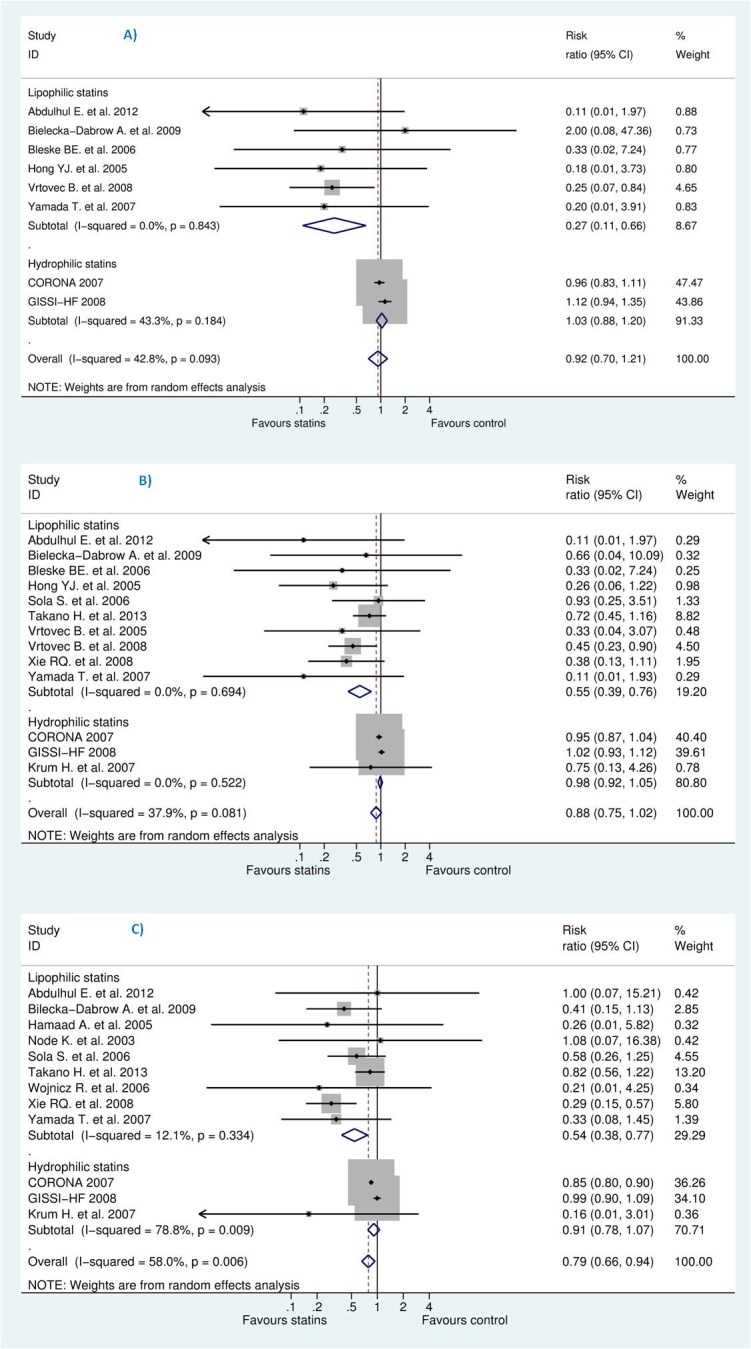
**Efficacy of statins compared with control in heart failure stratified by statin type (lipophilic versus hydrophilic) for the prevention of (A)** sudden cardiac death (SCD) **(B)** all-cause mortality, and **(C)** hospitalization for worsening heart failure (HWHF).

Although one cannot exclude that different statins would have different effects on HF outcomes, the hypothesis of differences among statins does not seem to be biologically plausible[16]. According to our sensitivity analysis, the inclusion or exclusion of CORONA [13] and GISSI-HF [12] impacted the result and changed it significantly. Also, since both studies represented 80% of the population in the analysis, we believe that only large RCTs with head-to-head comparison would give us reliable evidence (see Fig 12). Of note, two studies [36,39] directly compared atorvastatin (lipophilic statin) to rosuvastatin (hydrophilic statin) but for surrogate endpoints like C-reactive protein and hence did not evaluate any clinical outcomes with a similar output to *Lei Zhang et al*. [35,37].

On the other hand, a meta-analysis [33] evaluated statins’ effect in mortality prevention in heart failure with preserved ejection fraction (HFpEF) but included only cohorts and other observational studies. Our study, restricted to RCTs, did not include these patients. Similarly, a study [34] reported statin benefits in non-ischaemic cardiomyopathy and included two observational studies and ad-hoc analysis of 4 RCTs of which 2 were beta-blockers’ trials and so are not immune to bias. Although our analysis included 7 non-ischaemic HF trials, we could not pool them all together because of the limited data on mortality events.

A recent systematic review [19] concluded that statins did not decrease mortality, and might lead to little or no decrease in HWHF which is consistent with our findings. Likewise, a meta-analysis [20], included 13 trials, among the 24 trials in our analysis, concluded that statins might have no beneficial effects on all-cause death, cardiovascular death or pump failure and rehospitalization for heart failure in the overall (non-stratified) CHF populations. The authors stratified CHF patients according to age but since individual data were not used, the possible benefits for younger patients (< 65 years) might be unreliable as this could also be related to the stage and severity of CHF.

Of note, we think that the inability to prove the benefits of statins may be outweighed by a negative impact of cholesterol lowering in chronic heart failure (CHF) patients, as some evidence suggests that low levels of total cholesterol are associated with worse outcomes and a marked increase in mortality in CHF.[22,65–67]. Also any harm for statins in HF might also be masked, given the potential publication bias.

Overall, our updated review supports the current guidelines and do not recommend statins in patients with diagnosis of heart failure as this will avoid unnecessary prescriptions, overuse of care, and might help reluctant or hesitating physicians to make an evidence-based decision based on updated knowledge.

### Limitations and strength of this study

One principle of a meta-analysis is that included studies should be as much as similar to each other as possible. However, it is almost impossible to find identical studies with the same patient characteristics. This limitation is common and particularly in heart failure (HF) patients who usually have various characteristics and co-morbidities.

Our study revealed a potential for publication bias as shown from funnel plots for clinical outcomes. Although, this might be a serious limitation, we tried to take it into account in the study synthesis. As a consequence, this limitation had also downgraded the quality of the evidence from high to moderate within GRADE approach.

Our study had no access to individual data and unpublished studies and no data received by correspondence, though we have contacted studies’ corresponding authors.

For SCD outcome, for instance, due to the limited number of studies in ischaemic and non-ischaemic groups and potential heterogeneity, we could not investigate if statins have the same effects on ischaemic and non-ischaemic heart failure.

For blinding of investigators or outcome assessors in included trials, most studies had unclear risk but we believe this might have little impact due to weak subjectivity at least for all-cause mortality outcome in double blind trials.

The studies, in our analysis, had not recruited HF patients with preserved ejection fraction (HFpEF) and so this result might not be applicable to them.

Our study could contribute to the understanding of the existing discordant evidence in the evaluation of statins efficacy in clinical outcomes in heart failure with reduced ejection fraction which is revealed by heterogeneity, publication bias and small-study effects within clinical trials.

To our knowledge, this is the first analysis that evaluates the efficacy of statins for SCD prevention in HF patients; it helps bridge the gap of controversy towards statin benefits in important clinical outcomes rather than surrogate endpoints.

### Ongoing studies and perspectives

An ongoing study [https://clinicaltrials.gov/ct2/show/NCT01554592] is expected to resolve the issue of statin withdrawal in CHF patients. Future RCTs are still needed to: (i) compare lipophilic statins with hydrophilic statins. (ii) verify any class effect for statins and (iii) if any subpopulation of heart failure might benefit from statins in order to improve survival and reduce morbidity.

## Conclusion

Limited by a potential publication bias, and heterogeneity between studies, our systematic review and meta-analysis showed that statins do not decrease SCD or all-cause mortality. The benefits of statins regarding a possible decrease in hospitalization for worsening heart failure was not supported by estimated predictive intervals which means the expected treatment effects for a new brand trial. Physicians should follow the current guidelines of ACCF/AHA and not systematically prescribe statins for heart failure patients with left ventricular ejection fraction less than 45%. Ongoing and future trials should shed more light if any subpopulation might benefit from a particular statin.

### Summary for current knowledge

Heart failure has a high morbidity and mortality rate despite significant advances in therapy, diagnosis and management.

There is some evidence that low total cholesterol is associated with worse outcomes in (advanced) chronic heart failure, contrary to the general population.

Statin effects in sudden cardiac death prevention in heart failure is unknown and current discordant studies for other clinical outcomes like all-cause mortality are unresolved.

### Summary for this study outcome

This study delivers a clear message of no benefits of statins in HF patients in response to an existing controversy.

Publication bias and small-study effects offer a possible explanation to the observed discrepancies between trials, and between previous and our meta-analyses.

Within available data and potential publication bias, statins are ineffective in sudden cardiac death prevention and all-cause mortality reduction and may or may not slightly reduce hospitalization for worsening heart failure.

## Supporting information

S1 File search strategy equations and result. (PDF)

S2 File PRISMA Checklist for statins in heart failure. (DOCX)

S1 Table Number of events in statin group versus control group for each outcome. (DOCX)

## Abbreviations

AMI: Acute Myocardial Infarction
CHF: Chronic Heart Failure
DC: Dilated Cardiomyopathy
DCM: Dilated Cardiomyopathy
GRADE: Grading of Recommendation Assessment, Development and Evaluation
HF: Heart Failure
HFpEF: Heart Failure with preserved Ejection Fraction
HWHF: Hospitalization for Worsening Heart Failure
IDCM: Idiopathic Dilated Cardiomyopathy
LVEF: Left Ventricular Ejection Fraction
NA: Not Available
NICM: Non-Ischaemic Cardiomyopathy
NR: Not Reported
NYHA: New York Heart Association
PCI: Percutaneous Coronary Intervention
RCTs: Randomized Clinical Trials
SCD: Sudden Cardiac Death
WHO: World Health Organization
ACCF: American College of Cardiology Foundation
AHA: American Heart Association
