# Supplementary material for: No benefits of statins for sudden cardiac death prevention in patients with heart failure and reduced ejection fraction: A meta-analysis of randomized controlled trials
Source: PLoS One. 2017 Feb 6;12(2):e0171168. doi: 10.1371/journal.pone.0171168 (PMC5293250; doi:10.1371/journal.pone.0171168)
Supplement: S1 File — (PDF) [file pone.0171168.s001.pdf]

S1 : search strategy equations and result.

Databses: Ovid MEDLINE, Embase, and ALL EBM.

Date 28.04.2016

Database(s): **Ovid MEDLINE(R) In-Process & Other Non-Indexed Citations and Ovid MEDLINE(R)** 1946 to Present

Search Strategy: old final retrieval number : 244

#	Searches	Results	Annotations
1	exp heart failure/ or (((heart or cardia* or myocardial) adj3 failure*) or (((heart or cardia*) adj3 decompensation*) or ((heart or cardia*) adj3 insufficienc*))).mp.	169277	
2	exp Hypolipidemic Agents/ or (((anti-hyperlipidemic or antihyperlipidemic or antilip?emi* or antilipidemic or hypolip?emic or hypolipidemic or lipid depressing or lipid lowering) adj3 (agent* or drug*)) or (antilipidemics or statin*)).mp.	132003	
3	exp hydroxymethyleglutaryl-coenzyme A reductase inhibitor/ or hydroxymethyleglutaryl-co a reductase inhibitor.mp.	0	
4	hydroxymethyleglutaryl-co a reductase inhibitor.mp. [mp=title, abstract, original title, name of substance word, subject heading word, keyword heading word, protocol supplementary concept word, rare disease supplementary concept word, unique identifier]	0	
5	Atorvastatin*.mp. [mp=title, abstract, original title, name of substance word, subject heading word, keyword heading word, protocol supplementary concept word, rare disease supplementary concept word, unique identifier]	7359	
6	Fluvastatin*.mp. [mp=title, abstract, original title, name of substance word, subject heading word, keyword heading word, protocol supplementary concept word, rare disease supplementary concept word, unique identifier]	1803	
7	Lovastatin*.mp. [mp=title, abstract, original title, name of substance word, subject heading word, keyword heading word, protocol supplementary concept word, rare disease supplementary concept word, unique identifier]	5431	
8	Pravastatin*.mp. [mp=title, abstract, original title, name of substance word, subject heading word, keyword heading word, protocol supplementary concept word, rare disease supplementary concept word, unique identifier]	4382	
9	Rosuvastatin.mp. [mp=title, abstract, original title, name of substance word, subject heading word, keyword heading word, protocol supplementary concept word, rare disease	2667	

	supplementary concept word, unique identifier]	
10	Simvastatin*.mp. [mp=title, abstract, original title, name of substance word, subject heading word, keyword heading word, protocol supplementary concept word, rare disease supplementary concept word, unique identifier]	8840
11	((Hydrophilic or lipophilic) adj3 statin*).mp. [mp=title, abstract, original title, name of substance word, subject heading word, keyword heading word, protocol supplementary concept word, rare disease supplementary concept word, unique identifier]	276
12	review.ab.	899434
13	review.pt.	2100182
14	meta-analysis.ab.	63429
15	meta-analysis.pt.	64707
16	or/12-15	2479709
17	letter.pt.	911919
18	comment.pt.	660179
19	editorial.pt.	399757
20	or/17-19	1473826
21	16 not 20	2446741
22	exp Death, Sudden, Cardiac/ or Death, Sudden/ or exp heart arrest/ or (sudden adj3 (cardiac or death*)).mp. or (cardiovascular adj3 mortality).mp. or ((cardiac or cardiopulmonary or heart) adj3 arrest*).mp. or exp Mortality/ or mortality.mp. or mortality.fs.	1028465
23	or/2-11	134476
24	23 and 1	1904
25	24 and 21	551
26	25 and 22	247

Embase 28042016

Database(s): **Embase** 1974 to 2016 Week 17

Search Strategy: old final retrieval number : 818

#	Searches	Results	Annotations
1	exp heart failure/ or (((heart or cardia* or myocardial) adj3 failure*) or (((heart or cardia*) adj3 decompensation*) or ((heart or cardia*) adj3 insufficienc*))).mp.	412054	
2	exp Hypolipidemic Agents/ or (((anti-hyperlipidemic or antihyperlipidemic or antilip?emi* or antilipidemic or hypolip?emic or hypolipidemic or lipid depressing or lipid lowering) adj3 (agent* or drug*)) or (antilipidemics or statin*)).mp.	250602	
3	exp hydroxymethyleglutaryl-coenzyme A reductase inhibitor/ or hydroxymethyleglutaryl-co a reductase inhibitor.mp.	0	
4	hydroxymethyleglutaryl-co a reductase inhibitor.mp.	0	
5	atorvastatin*.mp.	29231	
6	fluvastatin*.mp.	2323	
7	lovastatin*.mp.	4614	
8	pravastatin*.mp.	17779	
9	Rosuvastatin.mp.	10810	
10	simvastatin*.mp.	32057	
11	((Hydrophilic or lipophilic) adj3 statin*).mp.	435	
12	meta-analy:.mp.	169859	
13	search:.tw.	368568	
14	review.pt.	2153932	
15	or/12-14	2502548	
16	exp Sudden Cardiac Death/ or Sudden Death/ or exp heart arrest/ or (sudden adj3 (cardiac or death*)).mp. or (cardiovascular adj3 mortality).mp. or ((cardiac or cardiopulmonary or heart) adj3 arrest*).mp. or Cardiovascular Mortality/	175812	
17	or/2-11	251435	
18	17 and 1	15072	
19	18 and 15	4427	
20	19 and 16	835	

All EBM 28042016

Database(s): **EBM Reviews - Cochrane Database of Systematic Reviews** 2005 to April 27, 2016**, EBM Reviews - ACP Journal Club** 1991 to April 2016**, EBM Reviews - Database of Abstracts of Reviews of Effects** 1st Quarter 2016**, EBM Reviews - Cochrane Central Register of Controlled Trials** March 2016**, EBM Reviews - Cochrane Methodology Register** 3rd Quarter 2012**, EBM Reviews - Health Technology Assessment** 1st Quarter 2016**, EBM Reviews - NHS Economic Evaluation Database** 1st Quarter 2016

Search Strategy: old final retrieval number : 82

#	Searches	Results	Annotations
1	exp heart failure/ or (((heart or cardia* or myocardial) adj3 failure*) or (((heart or cardia*) adj3 decompensation*) or ((heart or cardia*) adj3 insufficienc*))).mp.	17242	
2	exp Hypolipidemic Agents/ or (((anti-hyperlipidemic or antihyperlipidemic or antilip?emi* or antilipidemic or hypolip?emic or hypolipidemic or lipid depressing or lipid lowering) adj3 (agent* or drug*)) or (antilipidemics or statin*)).mp.	12344	
3	exp hydroxymethyleglutaryl-coenzyme A reductase inhibitor/ or hydroxymethyleglutaryl-co a reductase inhibitor.mp.	0	
4	hydroxymethyleglutaryl-co a reductase inhibitor.mp. [mp=ti, ab, tx, kw, ct, ot, sh, hw]	0	
5	Atorvastatin*.mp. [mp=ti, ab, tx, kw, ct, ot, sh, hw]	3360	
6	Fluvastatin*.mp. [mp=ti, ab, tx, kw, ct, ot, sh, hw]	651	
7	Lovastatin*.mp. [mp=ti, ab, tx, kw, ct, ot, sh, hw]	955	
8	Pravastatin*.mp. [mp=ti, ab, tx, kw, ct, ot, sh, hw]	1740	
9	Rosuvastatin.mp. [mp=ti, ab, tx, kw, ct, ot, sh, hw]	1234	
10	Simvastatin*.mp. [mp=ti, ab, tx, kw, ct, ot, sh, hw]	2753	
11	((Hydrophilic or lipophilic) adj3 statin*).mp. [mp=ti, ab, tx, kw, ct, ot, sh, hw]	52	
12	review.ab.	19697	
13	review.pt.	2559	
14	meta-analysis.ab.	5025	
15	meta-analysis.pt.	560	
16	or/12-15	24845	
17	letter.pt.	5894	
18	comment.pt.	1837	

19	editorial.pt.	342
20	or/17-19	6669
21	16 not 20	24807
22	exp Death, Sudden, Cardiac/ or Death, Sudden/ or exp heart arrest/ or (sudden adj3 (cardiac or death*)).mp. or (cardiovascular adj3 mortality).mp. or ((cardiac or cardiopulmonary or heart) adj3 arrest*).mp. or exp Mortality/ or mortality.mp. or mortality.fs.	55263
23	or/2-11	14816
24	23 and 1	661
25	24 and 21	102
26	25 and 22	82
