# Supplementary material for: No benefits of statins for sudden cardiac death prevention in patients with heart failure and reduced ejection fraction: A meta-analysis of randomized controlled trials
Source: PLoS One. 2017 Feb 6;12(2):e0171168. doi: 10.1371/journal.pone.0171168 (PMC5293250; doi:10.1371/journal.pone.0171168)
Supplement: S1 Table — (DOCX) [file pone.0171168.s003.docx]

| Outcome: Sudden cardiac death | | **Statin** | | | | | **Control** | | | | | |  |
| --- | --- | --- | --- | --- | --- | --- | --- | --- | --- | --- | --- | --- | --- |
| **Trial [Reference]** | | **Events** | | **Total** | | | **Events** | | | **Total** | | |  |
| Abdulhul E. et al. [40] | | 0 | | 28 | | | 4 | | | 28 | | |  |
| Bielecka-D. et.al [41] | | 1 | | 41 | | | 0 | | | 27 | | |  |
| Bleske BE. et al.[42] | | 0 | | 9 | | | 1 | | | 9 | | |  |
| CORONA [13] | | 316 | | 2514 | | | 327 | | | 2497 | | |  |
| GISSI-HF Investigators [12] | | 220 | | 2285 | | | 196 | | | 2289 | | |  |
| Hong YJ et al. [58] | | 0 | | 106 | | | 2 | | | 96 | | |  |
| Vrtovec B. et al. [54] | | 3 | | 55 | | | 12 | | | 55 | | |  |
| Yamada T. et al. [57] | | 0 | | 19 | | | 2 | | | 19 | | |  |
| Outcome: All-cause mortality | | **Statin** | | | | | | **Control** | | | | | |
| **Trial [Reference]** | | **Events** | | | **Total** | | | **Events** | | | **Total** | | |
| Abdulhul E. et al. [40] | | 0 | | | 28 | | | 4 | | | 28 | | |
| Bielecka-D. [41] | | 1 | | | 41 | | | 1 | | | 27 | | |
| Bleske BE. .[42] | | 0 | | | 9 | | | 1 | | | 9 | | |
| CORONA [13] | | 728 | | | 2514 | | | 759 | | | 2497 | | |
| GISSI-HF Investigators [12] | | 657 | | | 2285 | | | 644 | | | 2289 | | |
| Hong YJ et al.[58] | | 2 | | | 106 | | | 7 | | | 96 | | |
| Krum H. et al. [46] | | 2 | | | 40 | | | 3 | | | 45 | | |
| Sola S. et al. [51] | | 4 | | | 46 | | | 4 | | | 43 | | |
| Takano H. et al. [31] | | 27 | | | 288 | | | 37 | | | 286 | | |
| Vrtovec B. et al. [53] | | 1 | | | 40 | | | 3 | | | 40 | | |
| Vrtovec B. et al. [54] | | 9 | | | 55 | | | 20 | | | 55 | | |
| Xie RQ. et al. [56] | | 5 | | | 78 | | | 7 | | | 41 | | |
| Yamada T. et al. [57] | | 0 | | | 19 | | | 4 | | | 19 | | |
| **Table 3:** Number of events in statin group versus control group for each outcome  Outcome: Hospitalizations | **Statin** | | | | | **Control** | | | | | |  |  |
| **Trial [Reference]** | **Events** | | **Total** | | | **Events** | | | **Total** | | |  |  |
| Abdulhul E. et al. [40] | 1 | | 28 | | | 1 | | | 28 | | |  |  |
| Bielecka-D. et al. [41] | 5 | | 41 | | | 8 | | | 27 | | |  |  |
| CORONA [13] | 1109 | | 2514 | | | 1299 | | | 2497 | | |  |  |
| GISSI-HF Investigators [12] | 629 | | 2285 | | | 634 | | | 2289 | | |  |  |
| Hamaad A. et al. [44] | 0 | | 13 | | | 1 | | | 10 | | |  |  |
| Krum H. et al. [46] | 0 | | 40 | | | 3 | | | 45 | | |  |  |
| Node K. et al. [50] | 1 | | 24 | | | 1 | | | 26 | | |  |  |
| Sola S. et al. [51] | 8 | | 46 | | | 13 | | | 43 | | |  |  |
| Takano H. et al. [31] | 39 | | 288 | | | 47 | | | 286 | | |  |  |
| Wojnicz R. et al. [55] | 0 | | 36 | | | 2 | | | 38 | | |  |  |
| Xie RQ. et al. [56] | 10 | | 78 | | | 18 | | | 41 | | |  |  |
| Yamada T. et al. [57] | 2 | | 19 | | | 6 | | | 19 | | |  |  |
